# Endometrial immune profiling as a new tool for preconceptional assessment in patients with systemic autoimmune diseases

**DOI:** 10.3389/fimmu.2023.1334231

**Published:** 2024-01-05

**Authors:** Juan J. Fierro, Jelmer R. Prins, Svenja Henning, Hendrika Bootsma, Johanna Westra, Karina de Leeuw

**Affiliations:** ^1^ Department of Rheumatology and Clinical Immunology, University of Groningen, University Medical Center Groningen, Groningen, Netherlands; ^2^ Grupo Reproducción, Departamento de Microbiología y Parasitología, Universidad de Antioquia UdeA, Medellín, Colombia; ^3^ Department of Obstetrics and Gynecology, University of Groningen, University Medical Center Groningen, Groningen, Netherlands

**Keywords:** endometrial immune profiling, systemic lupus erythematosus, antiphospholipid syndrome, Sjögren's disease, pregnancy outcomes

## Introduction

Systemic autoimmune diseases such as systemic lupus erythematosus (SLE), antiphospholipid syndrome (APS), and Sjögren’s disease (SjD) affect predominantly women of childbearing age. SLE is a heterogeneous autoimmune disease characterized by interferon upregulation, production of autoantibodies and systemic symptoms (1). Patients with APS have thrombosis and/or pregnancy morbidity associated with persistent positivity of antiphospholipid antibodies (aPL) (2), whereas SjD is characterized by exocrine glandular lymphocytic infiltration, sicca symptoms, extra glandular manifestations such as arthritis, and sometimes presence of auto-antibodies, especially against SSA and SSB (3). These autoimmune diseases have been associated with a higher risk of adverse pregnancy outcomes (APO) like intrauterine fetal death, fetal growth restriction (FGR), preterm birth, low birth weight, and preeclampsia (2–4). Type I interferon signature and complement activation in peripheral blood have been linked to this increased APO risk (5–7).

The menstrual cycle is governed by a sophisticated interaction involving endometrial cells, immune cells, cytokines and sex hormones (8). It is known that the endometrial immune environment prepares to accept the embryo and facilitates implantation (9). Receptive endometrium is characterized by a pro-inflammatory response, complement activation, and an adequate interaction between extracellular vesicles, endometrial epithelial cells and the blastocyst (10). Therefore, an optimal balance between pro-inflammatory factors and the tolerogenic adaptive immune response in the endometrial tissue is pivotal for proper embryo implantation (11, 12).

Preconceptional disease activity can predict APO in patients with systemic autoimmune diseases, which stresses the importance of remission prior to conception. However, not all APO can be predicted by these disease-related factors (7, 13). Local immune changes in the endometrial environment might also contribute to this increased risk for APO. Endometrial immune profiling is a new method to analyze the immune cell distribution and cytokine production in endometrial tissue samples or menstrual blood. Different techniques are used such as multiparameter flow cytometry or gene expression analysis (14). It has already been suggested as a new screening strategy for personalized care for couples with repeated embryo implantation failures using assisted-reproductive therapy (ART) (15). In this paper, we describe the endometrial immune environment, the method of endometrial immune profiling and hypothesize that this technique might be a valuable tool to assess local immune changes that possibly play a role in development of APO in women with systemic autoimmune diseases.

## Endometrial immune environment

Immune cells in the endometrium/decidua are essential during implantation and placentation and immune imbalances have been associated with subsequent placental development failure (16). Uterine natural killer cells (uNK), CD4+ T regulatory cells and different subsets of CD68+ macrophages play a pivotal role in decidualization (maturation of the endometrium) and immune tolerance maintenance throughout pregnancy (12).

Early in pregnancy, uNK account for 70% of decidual lymphocytes and are involved in decidualization, trophoblast cell invasion, uterine vascular remodeling and immune tolerance (17). Notably, using a bioinformatics approach, it has been shown that impaired uNK function and defective endometrium maturation during early pregnancy preceded the development of preeclampsia (18). Decidua natural killer cells (dNK) express an inhibitor profile during gestation regulated by maternal HLA-C alleles through inhibitory receptors such as killer cell immunoglobulin-like receptors (KIRs), natural killer cell receptor NKG2A, and leukocyte immunoglobulin-like receptor B1 (LILRB1) (17). Interestingly, higher endometrial levels of transforming growth factor-beta (TGF-ß) have been associated with dNK-impaired maturation in patients with preeclampsia (19).

Other maternal leukocytes are also involved in decidualization processes and induce immune tolerance of the semi-allogenic fetus during early stages of pregnancy. It has been shown that levels of CD4+ CD25+ Foxp3+ T regulatory cells in peripheral blood fluctuate during the menstrual cycle, possibly preparing for pregnancy (20). During pregnancy, CD4+ T regulatory cells support maternal vascular adaptation and prevent placental inflammatory pathology (21). A lower number of these cells and/or impaired function have been associated with defective placentation and subsequent development of preeclampsia (22). The local T helper cell phenotype varies throughout pregnancy. During the implantation window, a Th1 response is crucial in inducing low-grade inflammation, immune cell recruitment, and tissue remodeling (23). Later on, production of IL-4, IL-5, IL-10 and IL-13 is promoted by a shift towards Th2 phenotype induced by decidual dendritic cells and the increased production of human chorionic gonadotropin (hCG), progesterone and estradiol during pregnancy (24). T helper cell imbalances with a predominant Th1 phenotype and a lower count of IL-10 producing CD8+ T cells have been associated with implantation failure and pregnancy loss (25). Interestingly, Th17 phenotype polarization and lower levels of Foxp3 T regulatory cells have been identified in blood from women with early onset preeclampsia compared with normotensive controls (26). Decidual CD8+ T cells are involved in antiviral protection and fetal tolerance due to an immunosuppressive phenotype through T-cell immunoglobulin mucin-3 (Tim-3), programmed cell death 1 (PD-1), and Cytotoxic T-Lymphocyte associated protein 4 (CTLA-4) inhibitory pathways. Notably, blockage of these pathways was correlated with fetal loss in animal models (27, 28).

A higher proportion of CD20+ B cells has been identified in decidua from patients with recurrent pregnancy loss compared to controls (29). Although B cells make up a small proportion of decidual lymphocytes, a regulatory B cell subset has been linked to fetal tolerance and Th1 suppression, preventing allogeneic responses against the fetus through IL-10 production (24).

Endometrial CD68+ macrophages also play an essential role in the menstrual cycle, restoring the endometrial integrity in preparation for pregnancy (30). Alterations in proportions of endometrial classical activated macrophages (M1) and alternatively activated macrophages (M2) have been described as predictors of implantation failure in patients under ART (31). Notably, a higher prevalence of CD163+ M2 type macrophages has been described during the proliferative phase in endometrial tissue, and was related to adverse implantation outcomes (31).

In conclusion, a complex immunological endometrial environment is linked to decidualization and subsequent healthy pregnancy development in the general population, and immune imbalances have been detected in patients with APO, especially preeclampsia, fetal loss and infertility. Nevertheless, there is no information concerning endometrial immune imbalances in patients with SLE, APS or SjD.

## Endometrial immune profiling: from reproductive medicine to systemic autoimmune diseases

Lédée et al. proposed endometrial immune profiling based on RT-qPCR analysis of endometrial biopsies, collected by aspiration during mid-luteal phase, focusing on CD56+ uNK levels, Interleukin-18/Tumor necrosis factor-like weak inducer of apoptosis (IL-18/TWEAK) and Interleukin 15/Fibroblast growth factor-inducible molecule (IL-15/Fn-14) mRNA ratios as key factors involved in uterine embryo receptivity (14, 15). The IL-18/TWEAK mRNA ratio reflects the Th1/Th2 balance and local angiogenesis, while the IL-15/Fn-14 mRNA ratio reflects uNK maturation. An overactive endometrial immune environment in biopsies during mid-luteal phase was established in the presence of high ratios or high uNK count, while low endometrial immune activation corresponded to low ratios and/or low uNK count (15).

This method was used in infertility patients as a preconception tool to assess suitability of the uterine immunological environment for embryo implantation, and results were used for personalized treatment decisions (32). Treatment modifications differed between patients classified as overactive and those with low endometrial immune activation levels, but overall an increase in live birth rate was achieved in analyzed and treated patients compared to non-analyzed (32). In brief, overactive patients received high-dose luteal hormonal support, oral estradiol supplementation, 20mg of corticosteroids, and vitamin E. At the same time, in those with low endometrial immune activation levels, an endometrial scratching or other local injury was performed and accompanied by hCG supplementation and standard-dose luteal hormonal support (32).

In subsequent studies by Lédée et al., higher pregnancy rates were achieved in patients under ART with a history of repeated implantation failures or recurrent pregnancy loss after endometrial immune profiling assessment (14). Patients were classified as having no dysregulation, low immune activation, over immune activation or mixed profile and received personalized treatment accordingly. Those patients with over-immune activation or mixed profile received immunotherapy with corticosteroids adjunction or low molecular weight heparin in case of resistance to corticosteroids. In contrast, those with low immune activation received endometrial scratching (14). Overall, some infertility patients undergo uterine immune dysregulations and immune profiling might aid in optimizing care and outcomes in these patients (14, 33).

Marron, et al. performed endometrial analysis of luteal-phase biopsies focusing on lymphocyte subsets with a flow cytometry panel assessing natural killer cells (NKs) subsets (uNK, peripheral NKs and T NKs), CD19+ B lymphocytes, CD8+ T lymphocytes, CD4+ T lymphocytes and their Th1, Th2, Th17 and regulatory subsets. Notably, higher concentrations of peripheral NKs (CD16+, CD56^dim^) and CD19+ B lymphocytes were identified in patients with a history of infertility or recurrent pregnancy loss compared to healthy controls (34). Furthermore, commercial endometrial immune profiling tests using endometrial tissue samples have been introduced in clinical practice for patients under ART (35). In those patients, a high expression of B-cell CLL/Lymphoma 6 (BCL6) by immunohistochemistry was identified as a marker for implantation failure or pregnancy loss (36).

Assessment of uNK, plasma cells, CD68+ macrophages, CXC-motif ligand 1 (CXCL1), CXC-motif receptor 2 (CXCR2), syndecan-1 and Vascular Endothelial Growth Factor A (VEGF-A) with immunohistochemistry has been used to map the endometrial immune environment in infertility patients with endometriosis. Interestingly, more macrophages were found in patients with endometriosis than those without and macrophage count was negatively correlated with uNK count in these patients (37).

So different techniques are described to assess the endometrium immune balance. We acknowledge that sample collection is an issue of matter since endometrial biopsy is an invasive procedure with inherent risks. Recent studies confirmed that menstrual blood collected during the first twenty-four hours of menstruation resemble the endometrial environment and can be used as an alternative, easier-to-get sample (38, 39). Recently, multiparameter flow cytometry panels have analyzed lymphocyte subpopulations from menstrual blood samples. Notably, subpopulations differed from peripheral blood samples and between patients with recurrent pregnancy loss and healthy controls (39, 40). Although it has been described that the cytokine profile of menstrual blood differed from the peripheral blood with a higher expression of IL-6, IL-1ß and CXCL8 in healthy controls, there is no information concerning cytokine profile in menstrual blood in patients with systemic autoimmune diseases (41). Therefore, we hypothesize that menstrual blood immunophenotyping might provide valuable insight prior to conception in those patients (34).

## Discussion

“The European League Against Rheumatism (EULAR) recommendations for women’s health and pregnancy in patients with SLE or APS” encourage risk stratification before pregnancy considering disease activity, comorbidities, autoantibody profile and medication use as an essential tool to improve pregnancy outcomes in these patients (13). Nevertheless, a high proportion of patients still develop APO. Although different pathologic mechanisms have been associated with APO development, the specific chain of events preceding APO is not fully understood. Moreover, associations between disease activity, and endometrial immune changes are unknown.

A better understanding of pathophysiology, including new valid biomarkers, developing clinical instruments for obstetric risk assessment and new personalized interventions are essential to improve pregnancy outcomes in these patients. Given its association with APO development, endometrial immune profiling might be a new tool for preconceptional assessment in patients with systemic autoimmune diseases such as SLE, APS and SjD.

**Figure 1:**
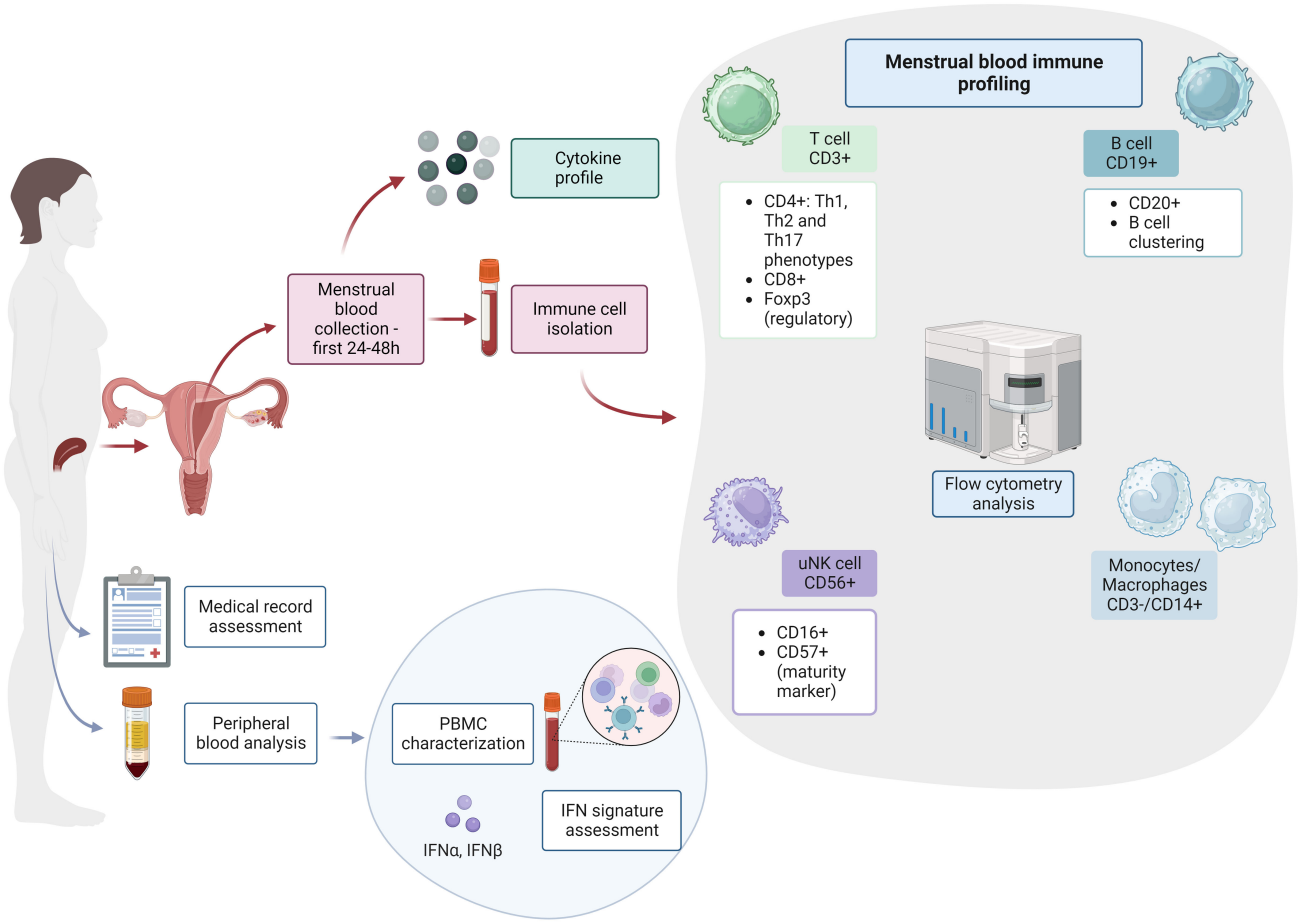
Endometrial immune profiling in patients with systemic autoimmune diseases. PBMC, peripheral blood mononuclear cell; IFN, interferon; T cell, T lymphocytes; B cell, B lymphocytes; uNK, uterine natural killer cells. This figure was created with BioRender.com.

We hypothesize that non-optimal endometrial immune imbalances might be related to APO in women with autoimmune diseases. In **Figure 1**, we propose a methodology combining endometrial immune profiling with assessment of clinical data, peripheral blood cells and interferon signature analysis for SLE, APS and SjD patients. We aim for a new approach combining previously described preconception risk factors and immunological changes in the endometrium (5, 7, 42). Briefly, we suggest assessing T cell, uNK, monocyte/macrophages and B cells in peripheral and menstrual blood as the principal immune cells involved in early placentation and widely linked with the pathogenesis of these autoimmune diseases (43, 44).

In conclusion, there is a lack of evidence regarding the endometrial immune environment in women with systemic autoimmune diseases in relation to APO. Due to the systemic pro-inflammatory state in these women, we expect an altered endometrial immune environment that can influence implantation and decidualization processes. The clinical value of endometrial immune profiling, in combination with previously used parameters for obstetric risk assessment, should be investigated in future studies. In particular, the use of menstrual blood seems to be a promising new and non-invasive technique, given the close resemblance with the endometrium biopsies.

## Author contributions

JF: Conceptualization, Methodology, Writing – original draft. JP: Writing – review & editing. SH: Writing – review & editing. HB: Writing – review & editing. JW: Writing – review & editing. KdL: Conceptualization, Writing – review & editing.
